# Endocarditis prevention: time for a review of NICE guidance

**DOI:** 10.1016/j.lanepe.2024.100876

**Published:** 2024-03-05

**Authors:** Martin Thornhill, Bernard Prendergast, Mark Dayer, Ash Frisby, Larry M. Baddour

**Affiliations:** a Unit of Oral & Maxillofacial Medicine, Surgery and Pathology, School of Clinical Dentistry, University of Sheffield, Sheffield, UK; b Guy's and St Thomas' Hospital, London and Chair of Cardiology, Cleveland Clinic, London, UK; c Somerset NHS Foundation Trust, Somerset, UK; d Patient Advocate, London, UK; e Division of Public Health, Infectious Diseases and Occupational Health, Departments of Medicine and Cardiovascular Medicine, Mayo Clinic College of Medicine, Rochester, MN, USA

**Keywords:** Infective endocarditis, Antibiotic prophylaxis, Invasive dental procedures, Guidelines, NICE

## Abstract

In 2023, the European Society of Cardiology (ESC) updated its infective endocarditis (IE) guidelines strongly endorsing antibiotic prophylaxis (AP) before invasive dental procedures (IDPs) for high-risk patients, elevating their recommendation to Class I. The American Heart Association (AHA) is aligned with this view and reaffirmed the need for AP to prevent IE in those at high-risk in its 2021 guidelines. In contrast, the UK's National Institute for Health and Care Excellence (NICE) recommends against routine AP use. Despite considerable new evidence, NICE has not reviewed this recommendation since 2015. In this Personal View, we review the new evidence that has arisen since 2015. Our analysis establishes the association between IDPs and IE and shows that AP is both safe and effective in reducing the IE-risk following IDPs in those at high-risk. Data also show that AP is cost-effective and would result in significant cost savings and health benefits if re-introduced into the UK's National Health Service for high-risk patients. Given these insights, we argue it is time NICE reviewed its guidance so that high-risk patients in the UK receive the same protection against IE that is afforded to patients in the rest of the world.

**Funding:**

The authors received no specific funding for this work.

## Introduction

The European Society of Cardiology (ESC) recently reviewed all available evidence in its 2023 guidelines for the prevention, diagnosis and management of infective endocarditis (IE) and concluded that high-risk patients should continue to receive antibiotic prophylaxis (AP) before invasive dental procedures (IDPs) to reduce the risk of IE.^1^ Moreover, based upon the strength of more current evidence since 2015, they have upgraded the recommendation from Class IIa (weight of evidence/opinion is in favour of usefulness/efficacy) to Class I (evidence and/or agreement that a given treatment or procedure is beneficial, useful, effective), and also indicated that “AP may be considered in patients at moderate risk on an individual basis” (Class IIb). Consistent with this guidance, the American Heart Association (AHA) also reviewed the available evidence in 2021 and concluded that high-risk patients should continue to receive AP before IDPs.^2^ Sixteen years after its recommendation that AP should not be used to prevent IE, we argue that the time has come for the UK National Institute for Health and Care Excellence (NICE) to review its position and align with international opinion.

IE is a devastating infection of the heart valves with a 30% first-year mortality^3^^,^^4^ whose incidence continues to increase in the UK,^5^, ^6^, ^7^ likely as a consequence of multiple factors including an ageing population, increasing numbers of high-risk individuals (due to medical interventions), increased awareness of IE and reduction in the use of AP before IDPs.

The AHA produced the first guidelines recommending the use of AP to reduce the risk of IE following IDPs in 1955.^8^ Although similar guidelines were adopted in the UK and the rest of the world, increasing concerns regarding the lack of evidence for AP efficacy, risk of adverse drug reactions (ADRs) and the development of antimicrobial resistance (AMR) led the British Society for Antimicrobial Chemotherapy (BSAC) to recommend in 2006 that AP use should be restricted to those at highest risk of IE (Table 1).^10^ This proposal was considered excessive by several UK cardiology organisations, and the issue was referred to NICE. There was, therefore, considerable surprise in 2008 when NICE recommended that AP use to prevent IE should cease completely.^12^ This conflicted with international guideline recommendations from the AHA (in 2007)^14^ and ESC (in 2009)^9^ who recommended that AP use should be restricted to those at the highest IE-risk undergoing IDPs. In October 2012, Sweden followed NICE and also recommended against the use of AP to prevent IE.^13^

**Table 1:** Cardiac conditions that identify individuals at high-, moderate- or low-risk of developing infective endocarditis.

High risk
Previous history of infective endocarditis
Presence of prosthetic cardiac valve (including transcatheter valves)
Prosthetic material used for valve repair (including annuloplasty and transcatheter valve procedures using prosthetic material)
Unrepaired cyanotic congenital heart disease
Congenital heart disease intervention using palliative shunts or conduits
Completely repaired congenital heart defect with prosthetic material or device (whether placed by surgical or transcatheter techniques)^a^
Patients with ventricular assist devices^b^
Antibiotic prophylaxis may be considered in heart transplant recipients^b^
Moderate Risk (also known as intermediate risk)
Rheumatic heart disease
Non-rheumatic valve disease (including mitral valve prolapse)
Congenital valve anomalies (including aortic stenosis)
Patients with cardiac implantable electronic devices (CIED) e.g. pacemaker or defibrillator^b^
Hypertrophic cardiomyopathy
Low risk
Patients with none of the above high- or moderate-risk conditions

**Notes:** Based on the AHA^2^^,^^14^^,^^15^ and ESC^9^^,^^11^^,^^16^ guideline definitions of those at high-, moderate- or low-IE-risk. IE, infective endocarditis.

a For the first 6 months after the procedure only.

b New moderate-and high-risk conditions added to 2023 ESC guidance.^1^

In 2015, an observational study found high compliance with the NICE guidance in the UK and identified an 88% decline in AP prescribing since 2008. It also found a significant increase in IE incidence following the change.^5^ At that time, NICE methodology required randomised controlled trial (RCT) evidence to support any change in recommendations and, following review, it reiterated its guidance stating that “*antibiotic prophylaxis against infective endocarditis is not recommended for people undergoing dental procedures*”.^17^ The ESC reviewed the same evidence in 2015 but continued to recommend AP for high-risk patients.^11^ In 2016, however, without explanation or review, NICE changed the wording of its guidance as follows: “*antibiotic prophylaxis against infective endocarditis is not* ***routinely*** *recommended for people undergoing dental procedures*”.^18^ Sweden again followed NICE by softening its guidance in 2016, and recommended against the routine use of AP unless advised by the patient's cardiologist or physician.^19^

The word ‘routinely’ made the UK guidance ambiguous and has caused confusion for dentists, cardiologists and patients. No information was provided about “routine” and “non-routine” circumstances, and so some cardiology centres (e.g. the Royal Brompton Adult Congenital Heart Centre) adopted ESC guidelines.^20^ The Scottish Dental Clinical Effectiveness Programme (SDCEP) produced implementation advice for dentists in 2018 (later approved by NICE and adopted by Chief Dental Officers of England, Scotland, Wales and Northern Ireland)^21^ advising that “The vast majority of patients at increased risk of IE will not be prescribed antibiotic prophylaxis. However, for a very small number of patients, it may be prudent to consider antibiotic prophylaxis (non-routine management) in consultation with the patient and their cardiologist or cardiac surgeon”. The patients for whom SDCEP suggested AP may be considered were identical to those recommended by the ESC and AHA (Table 1). However, dentists were advised to only consider AP if advised by the patient's cardiologist or cardiac surgeon and to “discuss the potential benefits and risks of prophylaxis for invasive dental procedures with the patient to allow them to make an informed decision about whether prophylaxis is right for them.” Neither NICE nor SDCEP, however, provided dentists (or doctors) with detailed information concerning the risks and benefits of AP, thereby leaving clinicians in the invidious position of being unable to inform patients and patients without the facts they need to make these important decisions.

Importantly, pivotal new evidence has emerged since 2015. Moreover, NICE has changed its methodology in two important respects: (1) accepting that the requirement for RCT evidence may be inappropriate when such evidence is unavailable or unrealistic (as is the case with AP prevention of IE)^22^^,^^23^; and (2) acknowledging that decisions should not be based solely on considerations relating to cost-effectiveness.^22^^,^^23^ In light of these developments, an updated review of NICE AP guidelines is overdue.

## New evidence

### Search strategy and selection criteria

References for this review were identified through searches of PubMed from 2015 until May 2023 with the search terms “endocarditis”, “prevention”, “antibiotic prophylaxis”, “guidelines”, and “dental procedures”. Articles were also identified through searches of the authors’ own files and the reference lists of identified papers (excluding publications in languages other than English). The final reference list was generated based on originality and relevance.

### Time trend studies

A recent systematic review of 18 European studies reported a doubling in IE incidence over the past two decades in Europe,^24^ in contrast to the US where IE incidence has remained comparatively stable.^25^ When trends data were weighted according to the population size of individual European countries, an increase in the annual incidence of 0.27 cases per 100,000 people was observed. Staphylococci and streptococci were the most common pathogens and in-patient mortality ranged from 14.3% to 17.5%.

The increase is likely due to a combination of factors that may differ from country to country, but include an increasingly elderly population, a rise in cardiovascular device implantation (increasing the number or proportion of those at high IE risk), easier access to diagnostic imaging, improved coding, increased use of illicit injection drug use, increased awareness of IE and possibly restrictions in the use of AP.

The role of changes in the use of AP on IE incidence is difficult to determine from time trend studies since most did not consider this variable and even those that did, were unable to determine whether specific dental procedures in individuals were covered by AP, or whether residual AP prescribing was focussed on those at highest IE risk. Thus, it is difficult to directly correlate changes in IE incidence with changes in AP prescribing. Indeed, increases in IE incidence were identified in countries where AP was restricted to those at high IE risk (e.g. the Netherlands^26^ and Germany^27^) as well as those where AP ceased altogether (e.g. the UK^5^, ^6^, ^7^ and Sweden^28^^,^^29^). Some of these studies showed an acceleration in IE incidence following changes in AP guidance, suggesting (but not proving) an association,^5^^,^^7^^,^^26^^,^^27^ while others found no evidence of a significant change in the trajectory of IE incidence.^6^^,^^28^^,^^29^

### Association between IDPs and subsequent IE

Since 2015, several studies have investigated whether there is an association between IDPs and the subsequent development of IE. Most have been underpowered to detect a significant association or were performed in countries where AP was still recommended (thereby obscuring any potential association). However, several large studies have identified a significant association, despite being undertaken in countries where AP was recommended for high-risk patients.

A large US study of patients with employer-provided medical and dental insurance cover used both case-crossover and cohort methodologies.^30^ Case-crossover analysis of 3774 IE patients identified a significant association between IDPs and the development of IE in the 30 days following the procedure for patients at high IE risk (OR 2.0, 95% CI 1.6–2.5, p = 0.002). This was strongest following dental extractions (OR 11.1, 95% CI 7.3–16.7, p < 0.0001) and oral surgical procedures (OR 50.8, 95% CI 20.8–124.0, p < 0.0001). The cohort study of almost 8 million patients also found that the odds of developing IE were significantly increased in the 30 days following extractions (OR 9.2, 95% CI 5.5–15.9, p < 0.0001) and oral surgical procedures (OR 20.2, 95% CI 11.2–36.7) in those at high-risk.^30^ Importantly, this study also showed that only 32.6% of IDPs performed in high-risk patients were covered by AP. Thus in 67.4% of high-risk patients any association between IDPs and subsequent IE was not potentially obscured by the use of AP.

In a very similar study of US Medicaid patients,^31^ case-crossover analysis of 2647 IE-cases also confirmed an association between IDPs and the development of IE in the subsequent 30 days in those at high risk. Again, this was particularly the case following extractions (OR 3.7, 95% CI 2.7–5.3, p < 0.005) and oral surgical procedures (OR 10.7, 95% CI 5.2–21.9, p < 0.0001). The 1.68 million patient cohort study also found an increased incidence of IE in the 30 days following IDPs in those at high IE risk, particularly after extractions (OR 14.2, 95% CI 5.4–52.1, p < 0.0001) and oral surgical procedures (OR 30.0, 95% CI 9.6–119.3).^31^ This study also showed that only 25.9% of IDPs were covered by AP in high-risk patients.

Although neither of these studies included identification of the IE pathogen, the occurrence of IE within 30 days of the procedure supports an association between the procedure and the subsequent occurrence of IE regardless of the causal organism. However, it is also possible that the reason for undertaking the IDP (e.g. poor oral hygiene, dental caries or dental alveolar abscess) could be responsible for the association rather than the procedure itself. In either scenario, the causal organism is more likely to be of oral cavity origin, i.e., oral viridans group streptococci (OVGS), HACEK organisms (*Haemophilus* spp., *Aggregatibacter actinomycetemcomitans, Cardiobacterium hominis, Eikenella corrodens,* and *Kingella kingae*)^32^ or some enterococcal species associated with oral infections,^33^ rather than staphylococci (that account for the majority of non-oral cavity related IE cases) or other non-oral organisms.

Interestingly, although both studies demonstrated a significant association between IDPs and subsequent IE, this association was much stronger for extractions and oral surgical procedures than for scaling—the latter being one of the most common IDPs. One possible explanation is that scaling is performed by dentists and hygienists to improve and maintain good oral hygiene. Patients who undergo regular dental scaling have much better oral hygiene than those who do not,^34^ and poor oral hygiene increases the likelihood of recurrent transient bacteraemias following daily activities such as tooth brushing, flossing and mastication that may lead to IE.^35^ Consistent with this, a recent large study showed that poor oral hygiene was a significant independent risk factor for the development of IE caused by oral bacteria.^36^ Hence, despite being an IDP, regular scaling may reduce the risk of IE by improving oral hygiene over the long term. In contrast, dental extractions and oral surgical procedures are more likely to be indicated in those who neglect their oral hygiene. However, prospective clinical trials are needed to properly quantify the long term endocarditis risk reduction benefits of improving the oral hygiene and dental health of moderate- and high-risk patients.

In 2017, a French study compared the incidence of IDP in the 3 months preceding IE in 73 cases with OVGS-IE and 192 controls with IE caused by other bacteria.^37^ Cases were significantly more likely to have undergone IDPs in the 3 months before developing IE (OR 3.3, 95% CI 1.2–9.3). A second French study included cohort and case-crossover methodologies and focused on high-risk patients with prosthetic valves. In the 138,876-patient cohort study there was no significant increase in OVGS-IE in the 3 months following IDPs. However, there was a significant association between IDPs and the development of IE in the case-crossover study of 648 patients with prosthetic valves who developed OVGS-IE (OR 1.7, 95% CI 1.1–2.6, p = 0.03) despite AP use in half of these procedures.^38^ The authors concluded that both studies suggested that “invasive dental procedures may be associated with oral streptococcal infective endocarditis although the magnitude of this association remains uncertain”.

A study using the Korean national database for patients with implanted cardiac electrical devices found that IDPs were associated with a significantly increased risk of IE (OR 1.75, 95% CI 1.48–2.05; p < 0.001) in a population where only 1.24% of IDPs were covered by AP.^39^ A Taiwanese self-controlled case study also identified a significant association between IDPs and IE (age-adjusted incidence rate-ratio 1.14, 95% CI 1.02–1.26) but did not report the proportion of IDPs covered by AP.^40^

Since AP has not been recommended in the UK since 2008, any association between IDPs and IE should be fully exposed. A nationwide case-crossover study was therefore attempted, but limitations in collecting community dental procedure data over the period prior to death or hospital admission resulted in the data being incomplete and unreliable.^41^ However, this issue did not apply to IDPs performed in the hospital outpatient setting where a significant association between dental extractions or surgical tooth removal and subsequent development of IE was confirmed (OR 2.1, 95% CI 1.2–3.8, p < 0.05).^42^ This study also calculated the additional number of IE cases that were likely to occur following IDPs in those at low (0.9/100,000 procedures, 95% CI 0.2–2.1), moderate (3.9/100,000 procedures, 95% CI 0.7–9.3) or high-risk (49.5/100,000 procedures, 95% CI 9.5–119.9).^42^

A nested case-crossover and case–control study performed in Sweden (where AP was not recommended between October 2012 and March 2016) was unable to confirm an association between OVGS-IE and IDPs in those at high risk.^43^ However, the sample size was small with only 240 cases in the case–control arm (of whom only 6 underwent extractions and 5 scaling procedures). Similarly, there were only 4 extractions and 4 scaling procedures in the 3-month case period and 7 extractions and 9 scaling procedures in the 6-month control period among 213 participants in the case-crossover study. The authors acknowledged that “a study with larger sample size could clarify whether there is a lack of association”. Indeed, to achieve this sample size, the authors included patients from the 42 month period when AP was not recommended^13^ as well as those from the 51-month period when AP was recommended for all Swedish patients at risk of IE undergoing IDPs (July 2008–October 2012) and the 22-month period when AP was recommended if advised by a patient's cardiologist (March 2016–January 2018).^19^ Even when AP was not recommended, the authors noted that AP prescriptions fell by only 41% suggesting that AP was used for 59% of IDPs in high-risk patients. It is possible, therefore, that use of AP obscured any association between IDP and IE and that this study was underpowered to detect a significant association. Indeed, given the high proportion of participants likely to have received AP, it could just as logically be concluded that the study demonstrated the effectiveness of AP in preventing IE rather than demonstrating a lack of association between IDP and IE, as the authors concluded.

In our opinion, when taken together, these studies support an association between IDPs and subsequent IE, particularly in high-risk patients. Furthermore, the data demonstrating a relative risk of IE after IDPs that is 4 and 50 times greater in those at moderate and high risk, respectively, than in those at low risk could be helpful when discussing relative risk with patients (as recommended by NICE and SDCEP).^42^

### Antibiotic prophylaxis (AP) efficacy

**Fig. 1:**
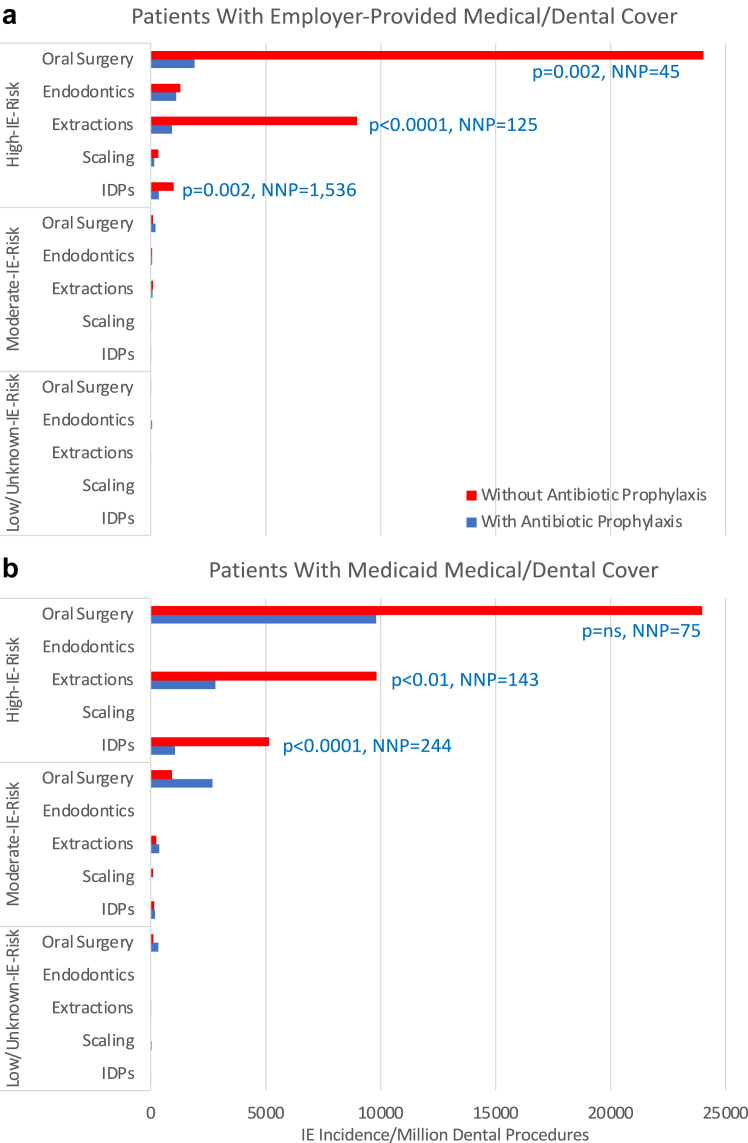
Infective endocarditis (IE) incidence in individuals at high-, moderate- or low-IE-risk following invasive dental procedures (IDPs), or IDPs of different types, performed with or without antibiotic prophylaxis cover. Study data from two different populations (a) patients with employer provided medical/dental cover^30^ (reproduced with permission from Elsevier) and (b) patients with Medicaid medical/dental cover^31^ (reproduced with permission from Wiley). Legend: p values compare IE incidence with and without AP cover (p = ns where no p value shown). NNP = number needed to prevent (i.e., the number of dental procedures that need AP cover to prevent one IE case). IE-risk status based on ESC and AHA guidelines (Table 1).

Only the two US studies mentioned above were able to identify whether specific dental procedures were covered by AP (or not) and thereby quantify the effect of AP on IE incidence. In high-risk patients with employer-provided medical/dental cover, AP significantly reduced IE incidence following IDPs (OR 0.4, 95% CI 0.2–0.6, p = 0.002), particularly extractions (OR 0.1, 95% CI 0.0–0.3, p < 0.0001) or oral surgical procedures (OR 0.1, 95% CI 0.0–0.4, p = 0.002; Fig. 1a).^30^ AP also significantly reduced IE-incidence following IDPs in high IE-risk Medicaid patients (OR 0.2, 95% CI 0.1–0.5, p < 0.0001), particularly extractions (OR 0.3, 95% CI 0.1–0.8, p < 0.01; Fig. 1b).^31^ To prevent one IE case, the number of IDPs, extractions or oral surgical procedures requiring AP cover (the number needed to prevent, NNP) was 1536, 125 and 45, respectively, for those with employer-provided medical/dental cover, and 244, 143 and 71, respectively, for Medicaid patients (Fig. 1).^31^

A recent systematic review and meta-analysis also highlighted the efficacy of AP in preventing IE, observing that no high-risk patients developed IE in studies where all such patients received AP before IDPs.^44^

These studies provide the best evidence to date that AP can significantly reduce the likelihood of IE following IDPs in those at high risk.

### Risk of adverse reactions (ADRs)

Soon after the 2015 review, new UK data demonstrated that the ADR risk following the standard 3 g dose of oral amoxicillin used for AP was substantially lower than NICE estimates.^45^ No fatal ADRs were reported after 3 million amoxicillin AP prescriptions and only 22.6 non-fatal ADRs/million prescriptions. Another UK study also recorded no deaths following amoxicillin AP.^46^

The NICE estimate of 20 fatal ADRs/million prescriptions^12^^,^^47^ relied on data from 1968^48^ and 1984,^49^ while the non-fatal ADR rate of 20,000/million prescriptions was derived from a 1997 estimate,^47^^,^^50^ leading them to conclude that “antibiotic prophylaxis against IE for dental procedures may lead to a greater number of deaths through fatal anaphylaxis than a strategy of no antibiotic prophylaxis, and is not cost-effective.”^12^ However, these estimates were for the ADR risk with all types of penicillin at all doses and via all routes of administration. Not surprisingly, therefore, the NICE estimates were much higher than the actual data for a single 3 g oral dose of amoxicillin used for AP purposes.

### Cost-effectiveness

A 2016 UK health economic analysis using ADR data specific to AP demonstrated that a reduction of only 1.4 high-risk IE cases per annum was required for AP to be cost-effective. The analysis also calculated that reinstating AP in England could achieve annual cost savings of £5.5–£8.2 million and health gains of >2600 quality-adjusted life-years.^51^

### Antimicrobial resistance (AMR)

AMR has the potential to deprive us of effective antibiotics to treat and prevent infections and is a major healthcare and public concern.^52^ Principal threats are the use of antimicrobials in food production, and their inappropriate use in veterinary and human healthcare.^52^ Antibiotic stewardship programmes aim to promote appropriate prescribing and emphasise the importance of limiting use to appropriate indications and prescribing the right antibiotic (i.e., with the right spectrum of activity to treat the infection) at sufficient dose (to effectively kill the organism while minimising side effects) for the shortest time (compatible with eradication of infection).

AMR is particularly a problem with infections due to *Escherichia coli, Staphylococcus aureus, Klebsiella pneumoniae and Streptococcus pneumoniae*.^53^ For OVGS infections, AMR is important, but fortunately less prevalent,^53^ although inappropriate use of amoxicillin to treat dentoalveolar infections merits particular attention. Amoxicillin is the most widely prescribed dental antibiotic, and despite guidelines recommending that dentoalveolar infections should be treated surgically (by extraction, endodontic treatment or incision and drainage), prescriptions to treat dental infections substantially exceed the use of a single high-dose of amoxicillin for AP purposes in most countries.^54^, ^55^, ^56^ Even in the presence of spreading infection, narrower spectrum penicillins, such as phenoxymethylpenicillin, are recommended in preference to amoxicillin by most guidelines. In addition to being inappropriate for many dental infections, amoxicillin is often prescribed at too low a dose for too long, further supporting the likelihood of AMR development.^54^, ^55^, ^56^ Therapeutic courses of amoxicillin can increase the selection of amoxicillin-resistant oral streptococci, although this effect is temporary and no longer significant after 28–35 days.^57^

Despite the use of high dose, short duration amoxicillin regimes for AP (single 2 g oral dose of amoxicillin in most countries; 3 g in the UK), there is evidence that this may temporarily increase the proportion of salivary amoxicillin-resistant OVGS for up to 5 days^58^ (although this appears to be less likely with a single 3 g oral dose of amoxicillin).^59^, ^60^, ^61^, ^62^ Indeed, the 3 g oral dose of amoxicillin was (and still is) recommended in the UK owing to the lack of effect on antibiotic sensitivity, increased effectiveness against OVGS, and minimal ADR.^10^^,^^21^^,^^59^, ^60^, ^61^, ^62^

Changing guidelines over the last 20 years have dramatically reduced the use of AP. Prior to the 2007 AHA guidelines,^14^ 2008 NICE guidelines^12^ and 2009 ESC guidelines,^9^ AP was recommended for those at both moderate and high risk of IE, and this remains the case in some parts of the world (e.g. Japan).^63^ There were three principal reasons why the AHA and ESC recommended cessation of AP for those at moderate risk, and why NICE recommended complete cessation: (1) the lack of evidence for AP efficacy (now addressed with new evidence—as discussed above); (2) the risk of adverse reactions (discussed above); and (3) concerns that unnecessary use of AP may contribute to the risk of AMR.^14^^,^^17^^,^^11^ Because the ratio of moderate to high-risk patients in the population is high (approximately 9:1),^64^ restricting AP to those at high-risk should reduce the number of individuals eligible for AP by ∼90%.^5^^,^^64^ Moreover, data showing that AP does not reduce the incidence of IE in moderate-risk individuals suggests that the recommendation to cease AP in this group was appropriate.^30^^,^^31^^,^^64^ However, dentists continue to prescribe AP inappropriately. In two recent studies, more than 80% of AP prescriptions were unnecessary.^65^^,^^66^ While in a study of US patients with employer-provided health insurance only 59,045 (2.8%) of 2,116,931 dental procedures covered by AP complied with AHA guidelines,^30^ and similarly, only 3470 (1.6%) of 218,258 dental procedures covered by AP complied with AHA guidelines in a study of Medicaid patients.^31^ Whilst some AP prescribing in low/unknown IE-risk US patients may be aimed at those with prosthetic joints (with a view to preventing prosthetic joint infections), there is no proven association between IDPs and prosthetic joint infections,^67^^,^^68^ and this practice is not supported by current US guidelines.^69^ Even in the UK, where there is no current dental indication for AP, 2292 dental prescriptions per month for a single 3 g oral dose of amoxicillin were still being issued by dentists 3-years after the change in guideline.^70^

There is therefore enormous scope for improved dental antibiotic stewardship measures to reduce the risk of AMR. The biggest opportunity is through better education and guidance concerning the management of dental infections. But there is also an opportunity to reduce the large amount of inappropriate AP prescribing and confine it to high-risk patients undergoing IDPs. More research and consideration of the best dose of amoxicillin (2 g or 3 g) might further reduce the risk of AMR. However, as suggested by the ESC and AHA guidelines, we argue that the benefits of AP outweigh the disadvantages when AP is confined to high-risk individuals undergoing IDPs.^1^^,^^2^ Moreover, the recent data demonstrating the effectiveness of AP^30^^,^^31^ suggests that eliminating AP use before IDPs in high-risk patients would significantly increase the number of preventable IE cases, and the consequent unnecessary mortality, morbidity, and need to use prolonged courses of antibiotic treatment to treat the condition (with their innate risk of promoting AMR).

## Impact in the UK

Currently, there are ∼397,000 high-risk individuals in the UK (0.6% of 67.3 million population) who undergo an average of 0.33 dental procedures annually (total 131,033).^51^^,^^71^ Of these, 48.5% (63,551) are IDPs,^41^ including 48,362 (76.1%) scaling procedures, 9978 (15.7%) extractions, 1398 (2.2%) endodontic treatments and 3813 (6%) surgical or mixed procedures.^41^ There is, therefore, considerable scope for AP to reduce IE incidence in the UK. The NNP data above suggest that ∼41–261 IE cases (including 12–78 deaths) could be prevented annually in the UK. Moreover, a cost-effectiveness analysis published in 2016 found that AP would be cost-effective in the UK if it prevented 1.4 high-risk individuals from developing IE each year.^51^ Therefore, the reintroduction of AP for high-risk individuals undergoing IDPs would not only prevent serious disease and save lives, it would also result in significant savings for the UK National Health Service.

## Conclusions and call for action

Most studies described herein have been published since the last NICE guideline review nine years ago. They establish the association between IDPs and IE and demonstrate that AP is safe, cost-effective, and efficacious. They also provide important new evidence that AP can reduce the IE risk for high-risk patients undergoing IDPs and support current ESC and AHA guidance. Whilst a well-designed RCT would be the ideal way to establish AP efficacy, this is unlikely to happen, and the large observational studies described here are as close as we are likely to come. In countries where guidelines recommend AP, randomisation of high-risk patients to placebo or no AP cover is regarded as ethically unacceptable due to the risk of developing IE (particularly with its attendant 30% first year mortality). Attempts to perform an RCT in the UK (where AP is not routinely recommended) have also failed due to the large numbers of high-risk patients that would need to be enrolled to achieve the statistical power required to detect a clinically significant effect, the difficulty of randomising these patients to AP or placebo in the thousands of dental practices across the country where IDPs are performed, and the consequent very high-cost of an RCT. Funders have felt unable to justify prioritising the high cost of funding an RCT of AP prevention of IE over the need to support other less expensive life-saving clinical research, particularly when compelling observational data on AP efficacy already exist.^72^

In our view, a review of NICE guidance is now essential so that high-risk patients in the UK can benefit from the same protection afforded these patients in the rest of Europe and the world. Ideally, NICE would adopt similar recommendations to those of the 2023 ESC guidance. If NICE recommended that clinicians in the UK adopt the ESC guidelines, then this would result in a uniform approach across Europe. Ultimately, the ESC and AHA (whose recommendations are very similar) could come together to universalise their IE prevention guidance (perhaps under the auspices of the WHO) so that patients and clinicians world-wide could benefit from a common, evidence-based approach to IE prevention that would minimise unnecessary antibiotic use and the development of bacterial resistance.

## Contributors

MT is a dentist and oral medicine specialist with research expertise in infective endocarditis and its relationship to the mouth. BP and MD are cardiologists with a specialist interest in infective endocarditis. AF is a patient advocate who lost her husband Myles to infective endocarditis that developed soon after a dental scaling in 2014 that was not covered by antibiotic prophylaxis. LB is an infectious diseases specialist with expertise in infective endocarditis prevention and treatment. All authors contributed to the content and writing.

## Patient involvement

A patient representative (AF) is a co-author of this paper and contributed to its content.

## Declaration of interests

MT reports research grant funding from the National Institutes for Health (USA), the British Heart Foundation, Delta Dental of Michigan Research and Data Institute's Research Committee and Renaissance Health Service Corporation (USA). BP reports receipt of expert testimony payments related to infective endocarditis and consultancy fees related to transcatheter heart valve procedures. MD reports expert testimony payments from Bevan Brittan, honoraria for presentations and support for attending meetings from Biotronik. AF reports no competing interests. LB reports consulting for Boston Scientific and Roivant Sciences, and royalty payments from UpToDate, Inc.

## References

[1] Delgado V., Marson A.N., de Waha S. (2023). 2023 ESC guidelines for the management of endocarditis: developed by the task force on the management of endocraditis of the European society of cardiology (ESC): endorsed by the European association of cardio-thoracis surgery (EACTS) and the European association of nuclear medicine (EANM). Eur Heart J.

[2] Wilson W.R., Gewitz M., Lockhart P.B. (2021). Prevention of viridans group streptococcal infective endocarditis: a scientific statement from the American Heart Association. Circulation.

[3] Ostergaard L., Voldstedlund M., Bruun N.E. (2022). Temporal changes, patient characteristics, and mortality, according to microbiological cause of infective endocarditis: a nationwide study. J Am Heart Assoc.

[4] Jensen A.D., Ostergaard L., Petersen J.K. (2022). Temporal trends of mortality in patients with infective endocarditis: a nationwide study. Eur Heart J Qual Care Clin Outcomes.

[5] Dayer M.J., Jones S., Prendergast B., Baddour L.M., Lockhart P.B., Thornhill M.H. (2015). Incidence of infective endocarditis in England, 2000-13: a secular trend, interrupted time-series analysis. Lancet.

[6] Quan T.P., Muller-Pebody B., Fawcett N. (2020). Investigation of the impact of the NICE guidelines regarding antibiotic prophylaxis during invasive dental procedures on the incidence of infective endocarditis in England: an electronic health records study. BMC Med.

[7] Thornhill M.H., Dayer M.J., Nicholl J., Prendergast B.D., Lockhart P.B., Baddour L.M. (2020). An alarming rise in incidence of infective endocarditis in England since 2009: why?. Lancet.

[8] Jones T.D., Baumgartner L., Bellows M.T. (1955). Committee on prevention of rheumatic fever and bacterial endocarditis, American Heart Association. Prevention of rheumatic fever and bacterial endocarditis through control of streptococcal infections. Circulation.

[9] Wilson W., Taubert K.A., Gewitz M. (2007). Prevention of infective endocarditis: guidelines from the American heart association: a guideline from the American heart association rheumatic fever, endocarditis, and Kawasaki disease committee, council on cardiovascular disease in the young, and the council on clinical cardiology, council on cardiovascular surgery and anesthesia, and the quality of Care and outcomes research interdisciplinary working group. Circulation.

[10] Dajani A.S., Taubert K.A., Wilson W. (1997). Prevention of bacterial endocarditis. Recommendations by the American heart association. JAMA.

[11] Habib G., Hoen B., Tornos P. (2009). Guidelines on the prevention, diagnosis, and treatment of infective endocarditis (new version 2009): the task force on the prevention, diagnosis, and treatment of infective endocarditis of the European society of cardiology (ESC). Endorsed by the European society of clinical microbiology and infectious diseases (ESCMID) and the international society of Chemotherapy (ISC) for infection and cancer. Eur Heart J.

[12] Habib G., Lancellotti P., Antunes M.J. (2015). 2015 ESC guidelines for the management of infective endocarditis: the task force for the management of infective endocarditis of the European society of cardiology (ESC)endorsed by: European association for cardio-thoracic surgery (EACTS), the European association of nuclear medicine (EANM). Eur Heart J.

[13] Horstkotte D., Follath F., Gutschik E. (2004). Guidelines on prevention, diagnosis and treatment of infective endocarditis executive summary; the task force on infective endocarditis of the European society of cardiology. Eur Heart J.

[14] Gould F.K., Elliott T.S., Foweraker J. (2006). Guidelines for the prevention of endocarditis: report of the working party of the British society for antimicrobial Chemotherapy. J Antimicrob Chemother.

[15] National Institute for Health and Care Excellence (NICE) (2008). http://www.nice.org.uk/guidance/cg64.

[16] Läkemedelsverket SMPA, (MPA) (2012).

[17] National Institute for Health and Care Excellence (NICE) (2015). http://www.nice.org.uk/guidance/cg64/chapter/Recommendations.

[18] National Institute for Health and Clinical Excellence (2008).

[19] Läkemedelsverket MPA (2016). https://www.lakemedelsverket.se/sv/behandling-och-forskrivning/behandlingsrekommendationer/sok-behandlingsrekommendationer/antibiotikaprofylax-i-tandvarden---behandlingsrekommendation.

[20] Tutarel O., Alonso-Gonzalez R., Montanaro C. (2018). Infective endocarditis in adults with congenital heart disease remains a lethal disease. Heart.

[21] Scottish Dental Clinical Clinical Effectiveness Programme (2018). http://www.sdcep.org.uk/published-guidance/antibiotic-prophylaxis/.

[22] Charlton V., Lomas J., Mitchell P. (2022). NICE's new methods: putting innovation first, but at what cost?. BMJ.

[23] NICE (2022). https://www.nice.org.uk/process/pmg36.

[24] Talha K.M., Baddour L.M., Thornhill M.H. (2021). Escalating incidence of infective endocarditis in Europe in the 21st century. Open Heart.

[25] Talha K.M., Dayer M.J., Thornhill M.H. (2021). Temporal trends of infective endocarditis in North America from 2000-2017- a systematic review. Open Forum Infect Dis.

[26] van den Brink F.S., Swaans M.J., Hoogendijk M.G. (2017). Increased incidence of infective endocarditis after the 2009 European society of cardiology guideline update: a nation-wide study in The Netherlands. Eur Heart J Qual Care Clin Outcomes.

[27] Keller K., von Bardeleben R.S., Ostad M.A. (2017). Temporal trends in the prevalence of infective endocarditis in Germany between 2005 and 2014. Am J Cardiol.

[28] Vahasarja N., Lund B., Ternhag A. (2020). Incidence of infective endocarditis caused by viridans group streptococci in Sweden—effect of cessation of antibiotic prophylaxis in dentistry for risk individuals. J Oral Microbiol.

[29] Vahasarja N., Lund B., Ternhag A. (2022).

[30] Thornhill M.H., Gibson T.B., Yoon F. (2022). Antibiotic prophylaxis against infective endocarditis before invasive dental procedures. J Am Coll Cardiol.

[31] Thornhill M.H., Gibson T.B., Yoon F. (2023). Endocarditis, invasive dental procedures, and antibiotic prophylaxis efficacy in US Medicaid patients. Oral Dis.

[32] Khaledi M., Sameni F., Afkhami H. (2022). Infective endocarditis by HACEK: a review. J Cardiothorac Surg.

[33] Komiyama E.Y., Lepesqueur L.S., Yassuda C.G. (2016). Enterococcus species in the oral cavity: prevalence, virulence factors and antimicrobial susceptibility. PLoS One.

[34] Smiley C.J., Tracy S.L., Abt E. (2015). Evidence-based clinical practice guideline on the nonsurgical treatment of chronic periodontitis by means of scaling and root planing with or without adjuncts. J Am Dent Assoc.

[35] Lockhart P.B., Brennan M.T., Thornhill M. (2009). Poor oral hygiene as a risk factor for infective endocarditis-related bacteremia. J Am Dent Assoc.

[36] Lockhart P.B., Chu V., Zhao J. (2023). Oral hygiene and infective endocarditis: a case control study. Oral Surg Oral Med Oral Pathol Oral Radiol.

[37] Duval X., Millot S., Chirouze C. (2017). Oral streptococcal endocarditis, oral hygiene habits, and recent dental procedures: a case-control study. Clin Infect Dis.

[38] Tubiana S., Blotiere P.O., Hoen B. (2017). Dental procedures, antibiotic prophylaxis, and endocarditis among people with prosthetic heart valves: nationwide population based cohort and a case crossover study. BMJ.

[39] Kim J.Y., Park S.J., Lee S.H., Seo G.H., Jang S.W. (2022). Risk of infective endocarditis associated with invasive dental procedures in patients with cardiac rhythm devices. Europace.

[40] Chen T.T., Yeh Y.C., Chien K.L., Lai M.S., Tu Y.K. (2018). Risk of infective endocarditis after invasive dental treatments: case-only study. Circulation.

[41] Thornhill M.H., Crum A., Rex S. (2022). Infective endocarditis following invasive dental procedures: IDEA case-crossover study. Health Technol Assess.

[42] Thornhill M.H., Crum A., Campbell R. (2023). Temporal association between invasive procedures and infective endocarditis. Heart.

[43] Vahasarja N., Lund B., Ternhag A. (2023). Oral streptococcal infective endocarditis among individuals at high risk following dental treatment: a nested case-crossover and case-control study. eClinicalMedicine.

[44] Lean S.S.H., Jou E., Ho J.S.Y., Jou E.G.L. (2023). Prophylactic antibiotic use for infective endocarditis: a systematic review and meta-analysis. BMJ Open.

[45] Thornhill M.H., Dayer M.J., Prendergast B., Baddour L.M., Jones S., Lockhart P.B. (2015). Incidence and nature of adverse reactions to antibiotics used as endocarditis prophylaxis. J Antimicrob Chemother.

[46] Lee P., Shanson D. (2007). Results of a UK survey of fatal anaphylaxis after oral amoxicillin. J Antimicrob Chemother.

[47] Agha Z., Lofgren R.P., VanRuiswyk J.V. (2005). Is antibiotic prophylaxis for bacterial endocarditis cost-effective?. Med Decis Making.

[48] Idsoe O., Guthe T., Willcox R.R., de Weck A.L. (1968). Nature and extent of penicillin side-reactions, with particular reference to fatalities from anaphylactic shock. Bull World Health Organ.

[49] Ahlstedt S. (1984). Penicillin allergy--can the incidence be reduced?. Allergy.

[50] deShazo R.D., Kemp S.F. (1997). Allergic reactions to drugs and biologic agents. JAMA.

[51] Franklin M., Wailoo A., Dayer M. (2016). The cost-effectiveness of antibiotic prophylaxis for patients at risk of infective endocarditis. Circulation.

[52] Ventola C.L. (2015). The antibiotic resistance crisis: part 1: causes and threats. P T.

[53] Antimicrobial Resistance C. (2022). Global burden of bacterial antimicrobial resistance in 2019: a systematic analysis. Lancet.

[54] Durkin M.J., Feng Q., Warren K. (2018). Assessment of inappropriate antibiotic prescribing among a large cohort of general dentists in the United States. J Am Dent Assoc.

[55] Teoh L., Stewart K., Marino R.J., McCullough M.J. (2018). Part 1. Current prescribing trends of antibiotics by dentists in Australia from 2013 to 2016. Aust Dent J.

[56] Thornhill M.H., Dayer M.J., Durkin M.J., Lockhart P.B., Baddour L.M. (2019). Oral antibiotic prescribing by NHS dentists in England 2010-2017. Br Dent J.

[57] Malhotra-Kumar S., Van Heirstraeten L., Coenen S. (2016). Impact of amoxicillin therapy on resistance selection in patients with community-acquired lower respiratory tract infections: a randomized, placebo-controlled study. J Antimicrob Chemother.

[58] Khalil D., Hultin M., Rashid M.U., Lund B. (2016). Oral microflora and selection of resistance after a single dose of amoxicillin. Clin Microbiol Infect.

[59] Shanson D.C., Ashford R.F., Singh J. (1980). High-dose oral amoxycillin for preventing endocarditis. Br Med J.

[60] Shanson D.C., Cannon P., Wilks M. (1978). Amoxycillin compared with penicillin V for the prophylaxis of dental bacteraemia. J Antimicrob Chemother.

[61] Simmons N.A., Cawson R.A., Clarke C. (1982). The antibiotic prophylaxis of infective endocarditis. Report of a working party of the British Society for Antimicrobial Chemotherapy. Lancet.

[62] Woodman A.J., Vidic J., Newman H.N., Marsh P.D. (1985). Effect of repeated high dose prophylaxis with amoxycillin on the resident oral flora of adult volunteers. J Med Microbiol.

[63] Nakatani S., Ohara T., Ashihara K. (2019). JCS 2017 guideline on prevention and treatment of infective endocarditis. Circ J.

[64] Thornhill M.H., Gibson T.B., Cutler E. (2018). Antibiotic prophylaxis and incidence of endocarditis before and after the 2007 AHA recommendations. J Am Coll Cardiol.

[65] Suda K.J., Calip G.S., Zhou J. (2019). Assessment of the appropriateness of antibiotic prescriptions for infection prophylaxis before dental procedures, 2011 to 2015. JAMA Netw Open.

[66] Suda K.J., Fitzpatrick M.A., Gibson G. (2022). Antibiotic prophylaxis prescriptions prior to dental visits in the Veterans' Health Administration (VHA), 2015-2019. Infect Control Hosp Epidemiol.

[67] Thornhill M.H., Crum A., Rex S. (2022). Analysis of prosthetic joint infections following invasive dental procedures in England. JAMA Netw Open.

[68] Thornhill M.H., Gibson T.B., Pack C. (2023). Quantifying the risk of prosthetic joint infections after invasive dental procedures and the effect of antibiotic prophylaxis. J Am Dent Assoc.

[69] Sollecito T.P., Abt E., Lockhart P.B. (2015). The use of prophylactic antibiotics prior to dental procedures in patients with prosthetic joints: evidence-based clinical practice guideline for dental practitioners--a report of the American Dental Association Council on Scientific Affairs. J Am Dent Assoc.

[70] Thornhill M.H., Dayer M.J., Forde J.M. (2011). Impact of the NICE guideline recommending cessation of antibiotic prophylaxis for prevention of infective endocarditis: before and after study. BMJ.

[71] Office for National Statistics (ONS) (2023). https://www.ons.gov.uk/peoplepopulationandcommunity.

[72] Thornhill M.H., Lockhart P.B., Prendergast B., Chambers J.B., Shanson D. (2015). NICE and antibiotic prophylaxis to prevent endocarditis. Br Dent J.

